# Remnant‐Preserving Posterior Cruciate Ligament Reconstruction Over Remnant Fibers Using a Figure‐of‐Four Position and a Posterior Trans‐Septal Portal

**DOI:** 10.1111/os.12755

**Published:** 2020-09-30

**Authors:** Yi‐lin Xiong, Chao Su, Shi‐da Kuang, Xin Zhao, Yu‐sheng Li, Wen‐feng Xiao, He‐yuan Zhu, Wei‐jie Liu, Shu‐guang Gao

**Affiliations:** ^1^ Department of Orthopaedics, Xiangya Hospital Central South University Changsha China; ^2^ Department of Orthopaedics Loudi Central Hospital of Hunan Loudi China; ^3^ Hunan Key Laboratory of Joint Degeneration and Injury Changsha China; ^4^ Hunan Engineering Research Center of Osteoarthritis Changsha China; ^5^ National Clinical Research Center of Geriatric Disorders Xiangya Hospital, Central South University Changsha China

**Keywords:** Arthroscopy, Posterior cruciate ligament, Reconstruction, Remnant preservation

## Abstract

Anatomic tunnel formation and remnant preservation are the recent trends in posterior cruciate ligament (PCL) reconstruction. However, it is difficult to observe the anatomical PCL footprint and perform the operation in the process of remnant‐preserving PCL reconstruction. This study describes a single‐bundle, transtibial PCL reconstruction technique with anatomic graft passage over the remnant PCL fibers. A femoral tunnel of PCL is created at 2 mm medial to the roof of the intercondylar notch and 3 mm proximal to the margin of the articular cartilage. The tibial insertion of PCL is observed using a figure‐of‐four position through a posterior trans‐septal portal. A tibial bone tunnel is made below the distal center portion of the tibial insertion of residual PCL fibers. The graft is passed over the PCL through the tibial bone tunnel, the space between the anterior cruciate ligament (ACL) and the residual PCL fibers, to the femoral socket and is fixed by the EndoButton and screw. This technique is able to ensure a reasonable intra‐articular length and optimal isometry. It has been applied in patients with PCL rupture and posterior instability of the knee joint, and no intraoperative or postoperative complications occurred. Our technology provides a valuable new treatment option for PCL rupture. Future comparative studies are needed to further clarify its beneficial effect.

## Introduction

Isolated posterior cruciate ligament (PCL) tear, often originating from sports or traffic trauma^1^, is an uncommon injury comprising only 1% of acute knee injuries^2^, ^3^. PCL is known to have an intrinsic ability to heal^4^, and good results have been reported for nonoperative treatment of PCL ruptures with mild to moderate instability^5^. However, long‐term follow‐up studies have revealed an increased risk of osteoarthritis development and functional impairment, making posterior cruciate ligament reconstruction (PCLR) more widely accepted when supported by evolving operating techniques^6^, ^7^.

In conventional PCLR techniques, the remnant fibers of PCL are generally removed to obtain a good visualization so as to facilitate the passage of a graft^8^, ^9^. Recently, many authors proposed the concept of preserving the remnant PCL fibers, including Humphrey and Wrisberg ligaments, during PCLR, which was deemed conducive to post‐operative knee stability, graft healing, and proprioceptive function^10^, ^11^, ^12^. Several studies have demonstrated that remnant preservation techniques enhanced the revascularization and regeneration of mechanoreceptors and achieved better activity‐related outcomes than techniques without remnant preservation^13^, ^14^, ^15^, ^16^.

However, applying arthroscopic techniques to perform a remnant‐preserving PCLR is challenging. Without a good visualization of the tibial attachment site, it can easily lead to malposition of the tibial tunnel, which seems to be one of the most important causes of failure^17^, and may even put posterior‐compartment neurovascular structures in danger^18^. In this paper, we intend to present a surgical procedure for arthroscopic PCLR with remnant preservation using a modified trans‐septal technique under visualization from the posteromedial portal in a figure‐of‐four position. This technique will help achieve good visualization of the tibial tunnel preparation, easy access to the tibial tunnel without neurovascular injury, and thorough preservation of remnant PCL fibers. The purpose of the present study is, therefore, to: (i) implement a novel method of remnant‐preserving PCL reconstruction; (ii) investigate its clinical and radiographic results; and (iii) discuss its outcome in the context of the existing literature.

## Methods

### *Inclusion Criteria*

Inclusion criteria for this study were: (i) grade III (laxity greater than 10 mm) PCL injury; (ii) patient treated with remnant‐preserving PCLR; (iii) patient aged between 18 to 45 years.

### *Exclusion Criteria*

Exclusion criteria for this study were: (i) multiple ligament injuries or bilateral injuries; (ii) subtotal meniscectomy or total meniscectomy at index surgery; (iii) combined fractures, malalignment or infection; (iv) neurovascular injury.

### *Patients Information*

**Fig. 1:**
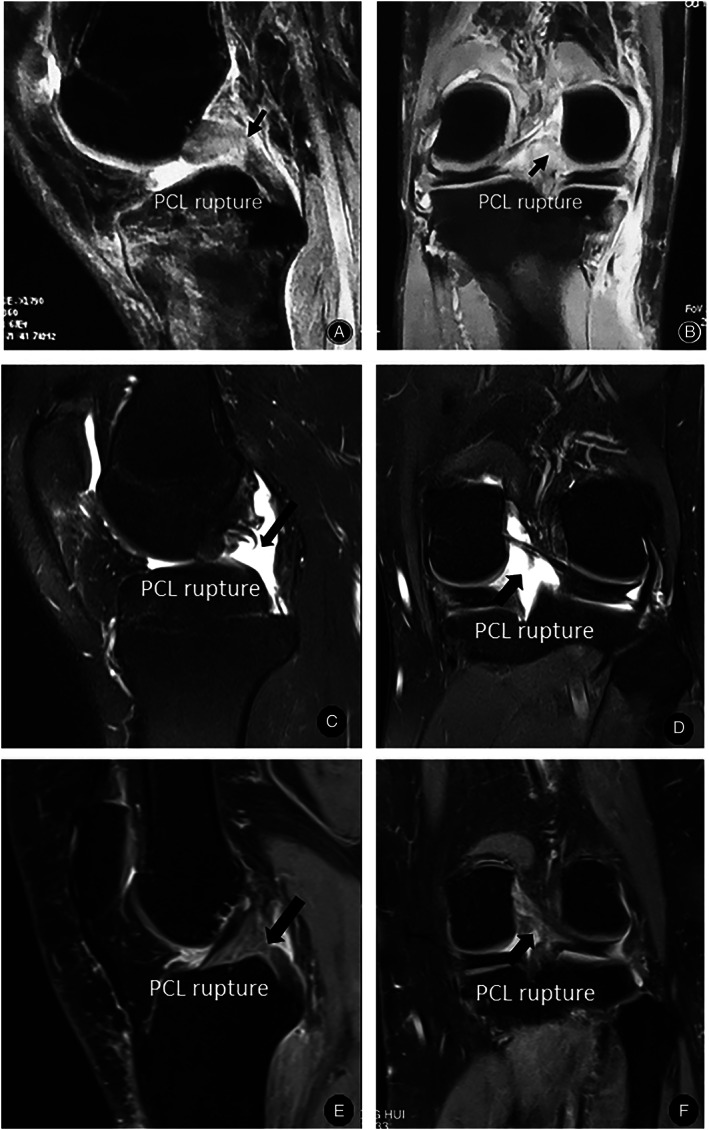
MRI examination of the knee in the reported three cases (A, B for Case 1; C, D for Case 2; E, F for Case 3): posterior cruciate ligament (PCL) rupture can be identified both in sagittal plane (A,C,E) and coronal plane (B,D,F).

Case 1 The patient, a 25‐year‐old male construction worker, broke his right knee when falling from his working spot at a height of 3 m 6 months ago. He was then sent to the nearest hospital immediately. The initial X‐ray examination ruled out any fractures around the knee, but later, MRI scan detected bone bruises of both tibia and femur, injuries of medial collateral ligament, patellar ligament and PCL, as well as knee effusion. The affected knee was immobilized in a plaster cast for 6 weeks, followed by rehabilitation for about 4 months. Thereafter, pain and swelling had largely resolved, while the laxity and weakness of the knee still persisted, accompanied by quadriceps atrophy. The patient was then admitted to our hospital with a diagnosis of PCL tear. Physical examination: slight swelling of the right knee, absence of local tenderness, positive posterior draw test, negative lateral stress test, negative rotation and extrusion test, and normal flexion and extension of the right knee joint; ROM (range of motion), F/E 125°/0°; MRI examination: right posterior cruciate ligament injury (grade III) (Fig. 1A, B).

Case 2 The patient, a 21‐year‐old male, injured his left knee in an accidental fall 2 weeks ago. He felt pain in the left knee and got a slight movement restriction of the injured knee. Physical examination: swelling of the left knee, absence of local tenderness, positive posterior draw test, negative lateral stress test, negative rotation and extrusion test; ROM, F/E 100°/0°; MRI examination: left posterior cruciate ligament injury (grade III) (Fig. 1C, D).

Case 3 The patient, a 34‐year‐old female, injured her left knee in a car accident 3 months ago. After the accident, she was then transferred to the local hospital, and treated conservatively for 3 months. But she still felt a swollen and painful left knee with limited range of motion. With a poor outcome of conservative treatment, she was referred to our hospital. Physical examination: slightly swelling of the left knee, local tenderness at the popliteal fossa, positive posterior draw test, negative lateral stress test, negative rotation and extrusion test; ROM, F/E 90°/0°. Posterior cruciate ligament rupture was identified by MRI examination (Fig. 1E, F).

### *Operative Procedure*

#### *Patient Setting and Graft Preparation*

Patient was placed in a supine position under regional anesthesia or general anesthesia. The tourniquet was applied to the thigh and inflated without elevation and exsanguination. Examination was performed under anesthesia to ensure a sufficient level of ligament laxity and a positive posterior drawer test. A routine arthroscopic examination was performed through the anterolateral and anteromedial portals. In order to facilitate the operation of instruments, the anteromedial portal was created adjacent to the patellar tendon and the inferior polar of patella. During the arthroscopic examination, the cruciate ligaments and both menisci were identified and the meniscal tears were repaired as indicated.

A longitudinal skin incision of 2 to 3 cm was made medial to the tibial tuberosity, and the hamstring tendons (semitendinosus and gracilis) were then harvested from the affected knee with a tendon stripper. The grafts were quadrupled, sized, and pretensioned; the average size of the graft was 8 mm (range, 7 to 10 mm).

#### *Femoral Tunnel Preparation*

**Fig. 2:**
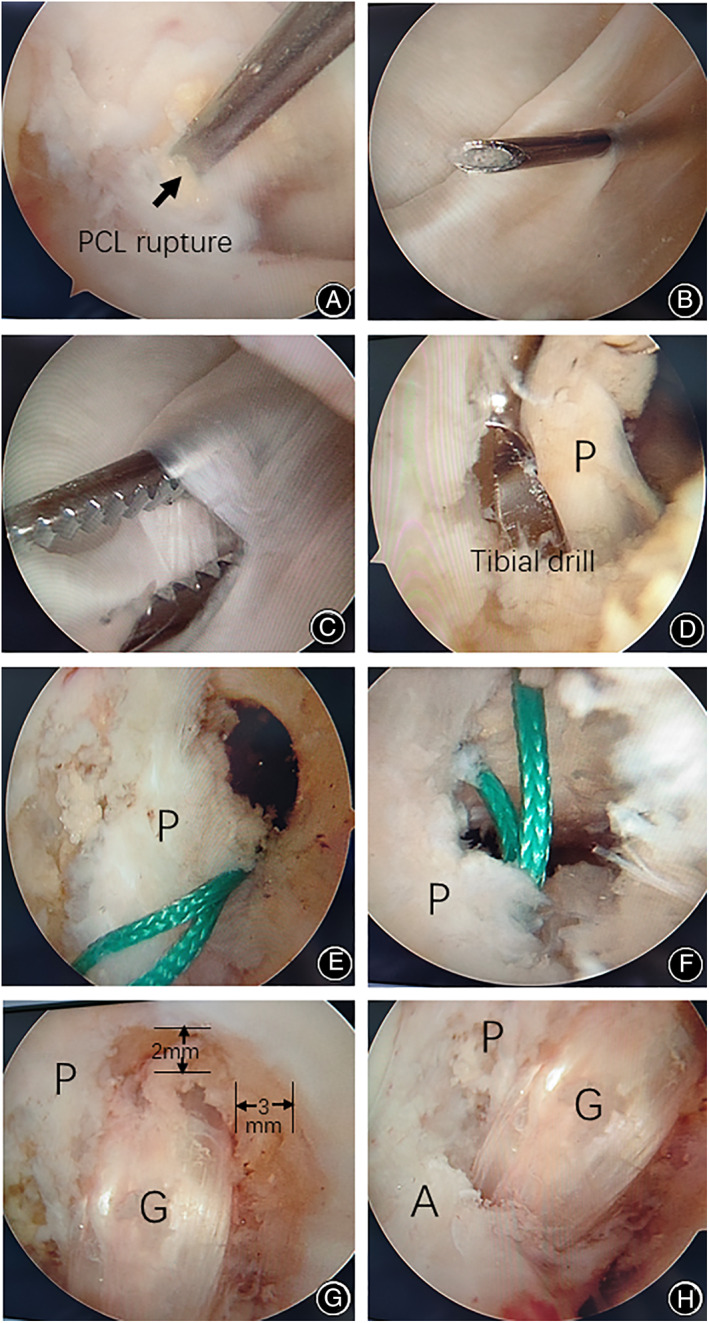
Surgical procedure under knee arthroscopy: (A) posterior cruciate ligament (PCL) rupture and remnant fibers were identified under arthroscopy. (B) the location of posteromedial portal was marked by spinal needle under direct visualization. (C) the posteromedial portal was enlarged with straight hemostatic forceps. (D) trans‐tibial tunnel was reamed alongside the PCL remnant (P) with neurovascular structures protected by a curette. (E) femoral tunnel was made with PCL remnant fibers (P) preserved, and the suture loop went through the top of PCL remnant fibers to the posterior compartment. (F) tibial tunnel was made with PCL remnant fibers (P) preserved, and the suture loop was at the lateral side of the distal part of residual PCL fibers. (G) graft (G) was passed through the femoral tunnel, which was created at 2 mm medial to the roof of the intercondylar notch and 3 mm proximal to the margin of articular cartilage. (H) graft (G) was fixed between PCL remnant (P) and anterior cruciate ligament (A).

The original PCL femoral insertion was identified at the medial femoral condyle under visualization through the anteromedial portal (Fig. 2A), and the footprint was marked by a radiofrequency device. The femoral tunnel was located about 6 to 7 mm from the trochlear point and the medial arch point of the medial femoral condyle, respectively (at 1 to 2 o'clock position on the right side or 10 to 11 o'clock position on the left side). With the knee in flexion, the femoral tunnel was created with a guide pin and a cannulated reamer. The bony tunnel depth was measured in order to identify a proper EndoButton loop length, which must ensure a minimum depth of 20 mm for graft placement. The femoral tunnel was reamed at a diameter of the graft size and to the calculated depth. All the bone dust and soft tissue were removed with a shaver. The PCL remnant on the femoral side should be preserved as much as possible during the whole process of tunnel preparation.

#### *Establishment of Trans‐Septal Posteromedial Portal*

**Fig. 3:**
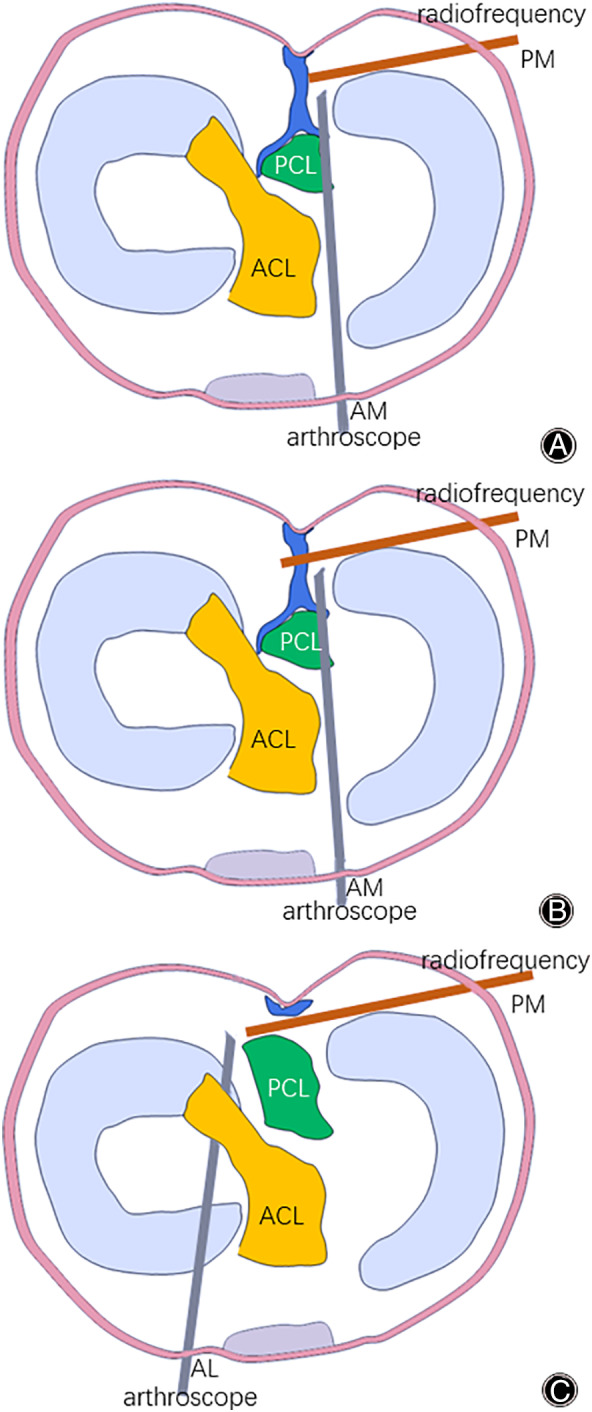
Surgical diagrams of establishing posterior trans‐septal portal ((A) arthroscope was placed in the space between the medial condyle and the posterior cruciate ligament (PCL) remnant from the anteromedial portal (AM) and the radiofrequency device passed through posteromedial portal (PM) to access the posteromedial compartment; (B) soft tissue of the posterior septum was carefully debrided with radiofrequency device; (C) arthroscope was redirected to the space between the lateral condyle and the anterior cruciate ligament (ACL) from the anterolateral portal (AL), and passed through to access the posterolateral compartment).

The arthroscope was redirected to and passed through the space between the medial condyle and the PCL remnant through the anteromedial portal to access the posteromedial compartment (Fig. 3A). Then, the affected leg was posed in a figure‐of‐four position to maintain the knee in a natural flexion about 90° by itself. Subsequently, the arthroscopic camera was turned to the medial wall of the capsule. After turning off the lights in the operating room, the transillumination spot could be visualized at the posteromedial aspect of the knee. Thereafter, the exact location for the posteromedial portal was marked by a spinal needle (Fig. 2B).

The posteromedial portal was created at the same point (Fig. 2C). The shaver and radiofrequency device were used for soft tissue debridement, and in order to avoid injury of the posterior neurovascular structure, the instruments should be operated as close to the bone as possible (Fig. 3B). Alternatively, a Wissinger rod could be used to make the trans‐septal portal. The Wissinger rod was inserted from the posteromedial portal, then slowly pushed through the posterior septum to the posterolateral compartment. After the establishment of trans‐septal posteromedial portal, the arthroscopic camera was inserted from the anterolateral portal, and then redirected to and passed through the space between the lateral condyle and the anterior cruciate ligament (ACL) to access the posterolateral compartment (Fig. 3C). The PCL remnant on the tibial side was then carefully detached from the posterior capsule under direct visualization, and the footprint was marked by the radiofrequency device.

#### *Tibial Tunnel Preparation*

The hook of the PCL tibial drill guide was introduced from the anteromedial portal through the space between the ACL and remnant PCL bundles and was further advanced into the PCL tibial footprint point as marked. Then, the arthroscope was switched to the posteromedial portal to provide a better visualization of the PCL tibial attachment site. If the drill guide was not accurately placed, appropriate adjustment could be made at this point of time. Then, a guide pin was introduced at the hamstring insertion site by placing the drill guide at an angle of 55° to 65°. In optimal cases, the pin should penetrate the middle or slightly lateral portion of the center of PCL remnant, approximately 1 cm below the joint line, and it is recommended to use a fluoroscopy device to confirm the position of the guide pin. After that, the tibial tunnel was made using a cannulated reamer of the graft size. During the process of drilling and reaming, particular care should be taken to avoid damaging the PCL remnant and to protect any neurovascular structures using a curette (Fig. 2D). Finally, the rasp was used to create a smooth acute angle at the anterior margin of the tibial tunnel.

#### *Graft Passage and Fixation*

The arthroscope was moved to the anteromedial portal, from which, a guide pin with a suture loop attached to its end was passed through the femoral tunnel out of the skin. The suture loop was left over just at the entrance of the femoral tunnel (Fig. 2E), and was then delivered to the posterior compartment using curved forceps. At the same time, a suture grasp instrument was introduced from the outlet of the tibial tunnel to grasp the suture loop (Fig. 2F). Then, the suture loop was pulled out from the tibial tunnel, and was used for retrieving the suture of the PCL graft through the bone tunnels. The PCL graft was slowly passed through the tibial tunnel and the femoral tunnel in the same manner until the EndoButton was flipped. Thereafter, the PCL graft was placed over the PCL remnant (Fig. 2G, H). After appropriate pretension, the PCL graft was fixed at the tibial site using an interference screw at a 60° knee flexion and anterior drawing position. The impingement and position of the PCL graft could be confirmed again from both the anterolateral and posteromedial viewing portals.

After completing the PCL reconstruction, instability of the joint was reassessed by the posterior drawer and posterolateral drawer tests. If a significant posterolateral translation was observed, it would be necessary to perform a posterolateral reconstruction.

## Results

**Fig. 4:**
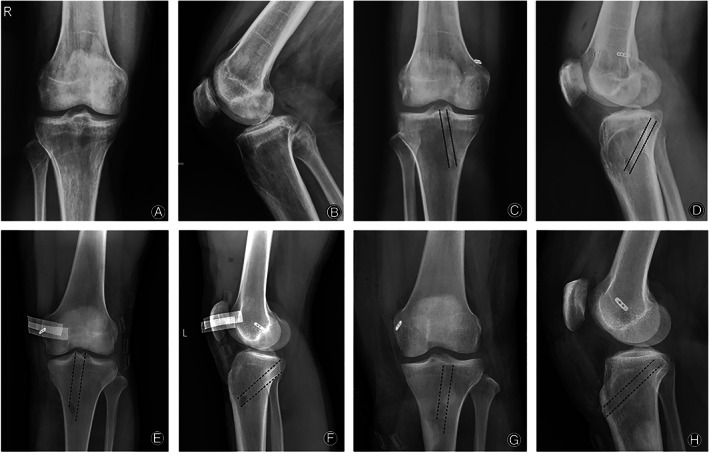
X‐ray examination of the patient knee joint pre‐ (A, B for Case 1) and after‐surgery (C, D for Case 1; E, F for Case 2; G, H for Case 3; dotted lines denoted the tibial tunnel).

X‐ray examinations were performed after the surgeries. Both anatomic tunnel and the correction of tibial posterior subluxation were identified on X‐rays (Fig. 4). No deep infection, thrombophlebitis, or neurovascular injury was noted in this study. After the operation, the patients underwent a rehabilitation process guided by a well‐designed rehabilitation protocol. When the patients were reexamined 3 months after operation, all the three patients could walk normally without braces or crutches. The postoperative ROM of the patients' knee joints were as follows (case 1–3): F/E, 130/0; 130/0; 140/0. Posterior drawer tests were all negative in these three patients. And there was no discomfort in the patients' daily activities.

## Discussions

When implementing a conventional transtibial PCL reconstruction, it is difficult to visualize the PCL remnant at the tibial attachment site. This can lead to malposition of the tibial tunnel^10^, ^16^, and cause impingement of the graft, resulting in graft loosening and failure. Additionally, poor visualization can also put neurovascular structures in danger^18^. In our technique, we used only the posteromedial portal to achieve excellent visualization, with preservation of the PCL remnant. As there is no need to involve the posterolateral portal or additional instruments, it is simpler to operate for junior surgeons and requires a shorter surgical time.

However, complication risk still exists in the posteromedial portal approach, since the saphenous nerve and vein are close to the posteromedial portal^19^. It is demonstrated that the posteromedial portal can be made in a safer way by positioning the knee in 90° flexion rather than in an extended position. The former position moves the saphenous nerve and vessels more posteriorly than the latter position does. The mean distance between the location of the posteromedial portal and the saphenous nerve is around 22–26 mm at a 90° flexion^20^. Given this, we proposed a figure‐of‐four position to hold the affected knee in a flexion about 90° by itself. This position makes the posteromedial portal approach much safer. Furthermore, when applying this position, the medial side of the knee turns upward, and therefore facilitates the operation of instruments.

**Fig. 5:**
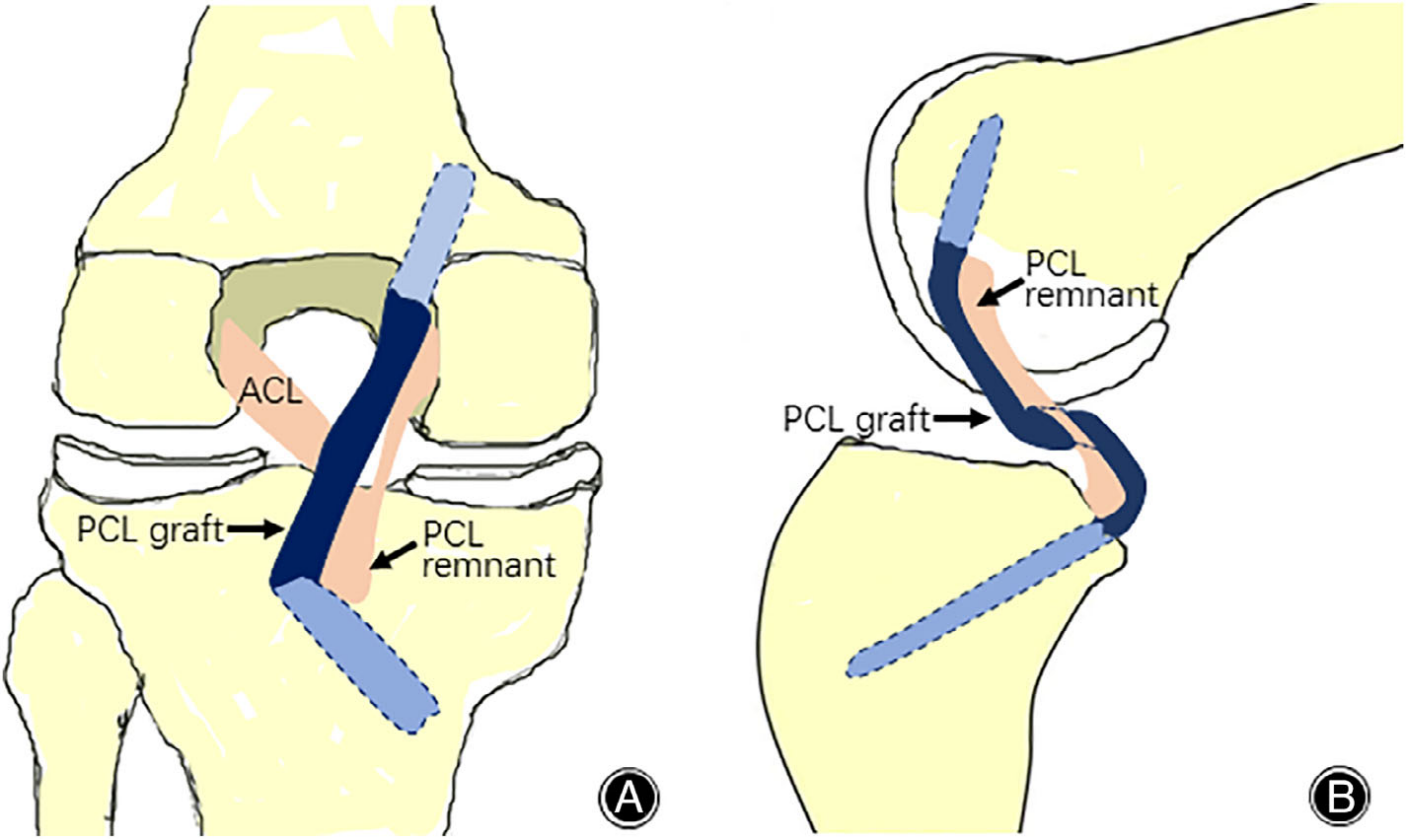
Schematic diagrams of remnant‐preserving posterior cruciate ligament reconstruction.

With the technique described in this study, a remnant‐preserving PCL reconstruction can be easily performed (Fig. 5). The excellent clinical outcomes of remnant‐preserving PCL reconstruction have also been confirmed by many other studies^11^, ^14^, ^15^, ^21^, ^22^, ^23^. For example, Lee *et al*.^11^ revealed that PCLR with remnant fiber preservation resulted in successful functional and morphological outcomes. They suggested that PCLR with remnant preservation could achieve less graft loosening in view of the reduction of the killer turn effect and the restoration of sensations for joint motion. Liu *et al*.^23^ followed up with 43 remnant‐preserved PCLR patients for an average of 38.4 months and reported that all clinical scores were improved significantly, with no complications observed. The results of KT‐1000 difference revealed a significant decline of posterior laxity, and the MRI evaluation confirmed no ligament retears. In addition, Ahn *et al*.^18^ showed that the remnant PCL fibers and the graft healed synchronously and formed a broad cross‐sectional area, and the graft was revascularized and healed with the remnant PCL fibers. Moreover, passing the PCL graft over the remnant of a previous PCL can increase the intra‐articular length. In this position, the PCL graft will have the least excursion and a good isometric function throughout the range of motion^24^.

### *Limitations*

One disadvantage of our technique is that, while creating the tunnels, we injured part of the original PCL insertion as a necessary step for anatomic tunnel placement. Another advantage is that the “killer angle” still exists, but this can be lessened by placing the knee in deep flexion when passing the graft, and we can also use a rasp to create a smooth acute angle at the anterior margin of the tibial tunnel. Finally, we only reported 3 cases in this study. The aim of this paper was not to provide the scientific foundation for our procedure, but rather to provide a newly modified approach to tackle the situation of PCL tear. In the future, more prospective studies are needed to determine the true value of this approach.

### *Conclusions*

Our technique illustrated a PCL reconstruction procedure using a figure‐of‐four position and a posterior trans‐septal portal, which enabled the creation of an accurate tibial tunnel without debriding the PCL remnant while protecting the neurovascular structures from injury. This modified remnant‐preserving PCL reconstruction technique may be recognized as a valid new treatment option for PCL tears in the future.

## Declaration

All authors contributed significantly and met the authorship criteria according to the latest guidelines of the International Committee of Medical Journal Editors. All authors agree to the final submitted manuscript.
